# The Immunomodulatory Role of Estrogen in Malaria: A Review of Sex Differences and Therapeutic Implications

**DOI:** 10.1002/iid3.70148

**Published:** 2025-02-03

**Authors:** Ye Wu, Ying‐Chun Chen, Fang‐Fang Liu, Ke Li

**Affiliations:** ^1^ Department of Pharmacy, Tongji Hospital, Tongji Medical College Huazhong University of Science and Technology Wuhan People's Republic of China; ^2^ Department of Laboratory Medicine, The Sixth Hospital of Wuhan Affiliated Hospital of Jianghan University Wuhan People's Republic of China; ^3^ Department of Pathology, The Central Hospital of Wuhan, Tongji Medical College Huazhong University of Science and Technology Wuhan People's Republic of China; ^4^ Department of Blood Transfusion, Tongji Hospital, Tongji Medical College Huazhong University of Science and Technology Wuhan People's Republic of China

**Keywords:** estrogen, GBD, immune system, malaria, sex differences, therapeutic target

## Abstract

**Background:**

Malaria remains a significant global health challenge, with substantial mortality rates, particularly in tropical and subtropical regions. A notable sexual dimorphism exists in malaria, with males often experiencing higher infection and mortality rates compared to females.

**Objective:**

This review explores the role of estrogen in modulating immune responses to malaria, potentially explaining the observed sex differences. Estrogen, through its receptors, influences immune cell activation and cytokine production, which are critical in the immune response to malaria.

**Results:**

Utilizing data from the Global Burden of Disease (GBD) study, we analyzed sex differences in malaria burden in Central Sub‐Saharan Africa from 2000 to 2021, revealing a significantly lower mortality burden for females compared to males. Epidemiological data and animal model results support the notion that estrogen plays a significant role in modulating immune responses to malaria. Estrogen receptors are widely expressed in immune cells, and estrogen can influence the activation, proliferation, and differentiation of these cells, thereby affecting cytokine production and immune response type. Additionally, selective estrogen receptor modulators (SERMs) show potential as therapeutic agents, with some studies demonstrating their efficacy in reducing parasitemia and improving malaria outcomes.

**Conclusion:**

Understanding the sex differences in the pathogenesis of malaria is crucial for its prevention, treatment, and vaccine development. Estrogen's role in immune regulation highlights the need for sex‐specific approaches in disease management.

## Graphical abstract

1. The present review highlights the significant sexual dimorphism in malaria, where males exhibit more severe symptoms and higher mortality rates compared to females, underscoring the need to incorporate gender factors in malaria research. 2. The present review emphasizes the role of estrogen in modulating immune responses to malaria, suggesting that understanding estrogen's impact could lead to novel therapeutic strategies and inform precision medicine approaches for the disease.

## 1 Introduction

Malaria, an ancient disease, continues to be among the most lethal parasitic illnesses in the world, presenting a substantial challenge to public health [1]. This disease leads to the infection of hundreds of millions of people annually, causing the death of hundreds of thousands, particularly in the tropical and subtropical regions of developing countries in Africa, Asia, and Latin America [2].

In the research and prevention of malaria, the impact of sex on the disease is an issue that is easily overlooked. There is a pronounced sexual dimorphism in malaria, with males often having higher rates of infection and mortality compared to females [3, 4]. However, the current state of affairs is such that many studies, especially basic research, are conducted on a single sex (mostly males), selectively ignoring the sex factor. Moreover, despite a considerable amount of epidemiological evidence, there are few reports on the differences in immune responses between males and females during malaria.

The gender differences in malaria can be attributed in part to the differences in levels of sex hormones [5, 6]. Among the sex hormones, estrogen has been more extensively studied. As the primary female sex hormone, estrogen not only plays a key role in the development and function of the reproductive system but also widely affects multiple systems in the body, including the cardiovascular, skeletal, and nervous systems [7]. In recent years, an increasing number of studies have revealed the important role of estrogen in regulating immune responses and inflammatory processes [8, 9]. Estrogen, by binding to its receptors, affects the activation, proliferation, and differentiation of immune cells, thereby regulating the production of cytokines and the type of immune response.

In light of the sex differences in malaria and the important role of estrogen in immune regulation, the present review focused on sexual dimorphism in malaria and the role of estrogen signaling in the disease. Here, we discussed the impact of estrogen on the function of immune cells, the association between changes in estrogen levels and the risk of malaria, and the potential applications of estrogen in the treatment and prevention of malaria. By thoroughly summarizing and concluding the associations between estrogen and malaria, we hope that this review can provide new perspectives for related basic research in the future.

## 2 Estrogen and Its Receptors

Estrogen refers to a class of female hormones that includes estrone (E1), estradiol (E2), estriol (E3), and estretrol (E4). Among them, E2 is the main circulating estrogen in humans. Since all the above four hormones have 18 carbons, they are referred to as C18 steroids [10]. Estrogen is mainly synthesized in the ovaries, but also the adrenal glands, bones, skin, and adipose tissue. In addition, neurons and astrocytes in the brain can also synthesize estrogen [11].

There are three different receptors for estrogen: estrogen receptor α (ERα), estrogen receptor β (ERβ), and G protein‐coupled estrogen receptor 1 (GPER1). ERα and ERβ belong to a family of ligand‐regulated transcription factors called steroid hormone receptors, while GPER1 has no transcriptional activity [12]. There are differences in the expression levels and patterns of these three receptors in various tissues and cells throughout the body, which also leads to distinct responses of different tissues and cells to estrogen. Human ERα and ERβ are encoded by ESR1 and ESR2 genes located on chromosomes 6 and 14, respectively. The downstream signaling pathways of estrogen mediated by ERα or ERβ are very complex, including genomic and non‐genomic effects [13]. There may be complementary or antagonistic effects between the two in different tissues or cells. We have observed this phenomenon in Alzheimer's disease that ERα and ERβ have Opposite effects on tau pathology [14]. in the T47D cell line with inducible expression of ERβ, analysis of ERα‐ and ERβ‐mediated gene regulation revealed that ERβ predominantly counteracted the effects of ERα on gene expression, inhibiting approximately 998 of the genes regulated by ERα, including those involved in cell proliferation and metabolism [15]. Another study demonstrates that in the same cellular context, ERα and ERβ homodimers regulate distinct transcriptomes and functions, with ERα promoting cell proliferation and ERβ exerting antiproliferative effects, influencing cell migration and modulating the expression of specific genes [16]. Owing to the extensive distribution of estrogen targets in the human body, there may also exist such antagonistic pathophysiological mechanisms for immune responses in bone marrow or peripheral blood cells of malaria patients, which is mediated by ERα and ERβ.

## 3 Effects of Estrogen on the Immune System

Estrogen receptors, particularly ERα, are expressed on the surface of various immune cells, such as T cells, B cells, macrophages, and dendritic cells [17, 18, 19, 20, 21, 22]. Consequently, the impact of estrogen on the immune system is multifaceted, involving the influence on the development, differentiation, cytokine secretion, and immune responses of multiple immune cells [8]. At the molecular level, it also involves interactions with other transcription factors like NF‐κB [23]. These effects are macroscopically manifested as different immune responses between genders when faced with external stimuli [24]. This section attempts to provide a brief summary and overview.

### 3.1 The Influence of Estrogen on T and B Cells

Estrogen affects the proliferation and differentiation of T cells, particularly the balance between helper T cells (Th1 and Th2) [25, 26, 27]. Th1 cells primarily produce interleukin‐12 (IL‐12), interferon‐γ (IFN‐γ), and tumor necrosis factor‐α (TNF‐α), participating in cell‐mediated immune responses and inflammatory reactions. In contrast, Th2 cells mainly produce interleukin‐4 (IL‐4), interleukin‐10 (IL‐10), and transforming growth factor‐β (TGF‐β), engaging in humoral immunity and allergic reactions. Estrogen tends to inhibit Th1 responses while promoting Th2 responses, which can explain why estrogen suppresses and enhances diseases mediated by Th1 and Th2, respectively. For instance, women are more susceptible to certain autoimmune diseases and allergic reactions, such as asthma [28] and systemic lupus erythematosus [29, 30]. Regarding B cells, studies have shown that estrogen can promote B cell proliferation and antibody production [31, 32, 33, 34].

### 3.2 The Influence of Estrogen on Macrophages and Dendritic Cells

It was reported that Estrogen can affect the polarization of macrophages, thereby regulating their roles in inflammation and tissue repair [35]. M1‐type macrophages are mainly involved in inflammatory responses, while M2‐type macrophages participate in anti‐inflammatory processes and tissue repair. Estrogen tends to promote the polarization of M2‐type macrophages. Animal experiments with viral infections have shown that female mice have a significantly higher number of macrophages in the pleural and peritoneal cavities compared to males, exhibiting stronger phagocytic capabilities and higher TLR expression levels, which are also associated with stronger acute inflammatory responses [36]. Dendritic cells are one of the important antigen‐presenting cells, and estrogen can regulate T cell activation and differentiation by affecting their development and functions [37, 38]. Additionally, it has been reported that in mouse bone marrow, estrogen (mainly estradiol) can upregulate IRF4 by binding to ERα in myeloid progenitor cells, thus promoting the differentiation of dendritic cells [39]. Estrogen can also influence the secretion of related cytokines in dendritic cells, such as promoting the expression of TNF‐α and IL‐1β, but does not affect the expression and secretion of IL‐10 [40].

### 3.3 The Influence of Estrogen on Natural Killer (NK) Cells

Estrogen can also affect the activity of NK cells, which are crucial innate immune cells. In the early stages of encountering viral infections, they can influence the immune system by producing IFN‐γ, thereby controlling viral load [41]. Evidence has shown that estrogen can affect the function of NK cells by regulating their proliferation and cytokine secretion [42]. Moreover, studies have shown that young men have a higher number of NK cells and cytotoxic activity in their blood compared to young women [43], but this gender difference is reversed in old age [44]. Since estrogen levels drop significantly after menopause in women, this age‐related reversal effect is likely due to estrogen deficiency.

### 3.4 The Influence of Estrogen on Neutrophils and Eosinophils

Studies have shown that the number of neutrophils in women's blood increases during pregnancy and the luteal phase of the menstrual cycle, indicating that higher levels of estrogen or progesterone may promote an increase in neutrophil numbers [45]. Additionally, evidence suggests that estrogen can delay neutrophil apoptosis by reducing the expression of the proapoptotic caspase 3 [46]. For eosinophils, in vitro experiments demonstrate that estrogen can increase the migration, adhesion, survival, and degranulation of eosinophils [47]. Furthermore, animal experiments have confirmed that the number of eosinophils in the uterus of female rats peaks during the estrous period when estrogen levels are high, and ovariectomy significantly reduces the number of eosinophils [48]. Similar to the observations in the uterus, estrogen treatment also increases the number of eosinophils in the blood and respiratory tract, and this effect is mediated by ERα [49].

Apart from the aforementioned immune cells, some studies have reported the effects of estrogen on other immune cells, such as monocytes [50], other T cell subsets or subtypes (e.g., innate γδ T cells, NKT cells) [51, 52], microglia [53] and astrocytes [54] in the brain, highlighting the broad impact of estrogen on the immune system. Estrogen's influence is not limited to these cell types but extends to the entire immune system, where it modulates the function and activity of various immune cells. This is particularly evident in the context of sex differences in immune responses, where females generally exhibit a stronger immune reaction compared to males [24, 29, 55, 56, 57].

The robustness of the female immune response is attributed in part to the influence of estrogen. Estrogen's effects are multifaceted, impacting both the innate and adaptive arms of the immune system [8, 58]. It promotes a more vigorous pro‐inflammatory response, affects the activity of NK cells, and influence the production of antibodies, which are all components of the innate and humoral immunity. Additionally, estrogen has been shown to influence the differentiation and function of T cells, which are central to cell‐mediated immunity. The hormone's immunomodulatory actions contribute to the observed sexual dimorphism in immune responses, where females mount a more effective defense against various pathogens [29].

By influencing immune cells, estrogen plays a significant role in maintaining immune homeostasis and defending against pathogen invasion. However, these effects of estrogen may also be subject to individual differences, fluctuations in hormone levels, and other environmental factors, which can modulate the immune response [59]. The interplay between estrogen and the immune system is complex and can lead to a heightened immune response in females, which, while beneficial for pathogen clearance, can also lead to increased risk of immunopathology in certain conditions, such as in hormone‐dependent breast cancer (estrogen and progesterone receptor positive) [60]. Understanding this relationship is crucial for developing a comprehensive view of how sex hormones influence immune responses and for designing sex‐specific therapeutic strategies.

## 4 The Global Distribution of Malaria and Epidemiological Evidence of Sex Differences in Malaria

**Figure 1:**
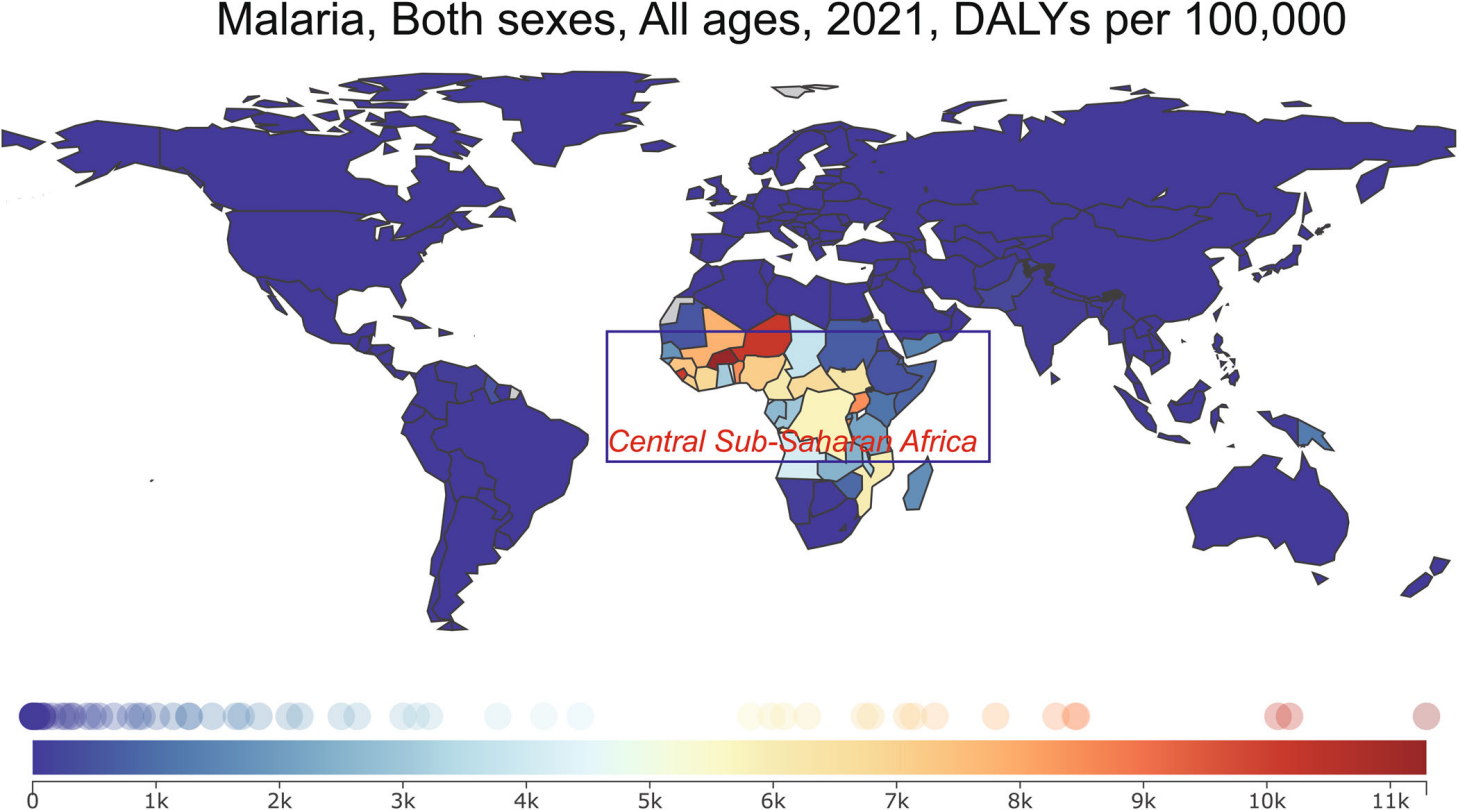
Geographical distribution of malaria burden in 2021. This map presents the data from the Global Burden of Disease (GBD) study (https://www.healthdata.org/), illustrating the distribution of malaria across the globe with a focus on disability‐adjusted life years (DALYs) per 100,000 people. The highest burdens are observed in central sub‐Saharan Africa, particularly in countries such as Mali, Niger, Nigeria, the Central African Republic, South Sudan, Chad, Uganda, and the Democratic Republic of Congo. The visualization demonstrates the regional concentration of malaria cases, with the majority of other global regions showing significantly lower or negligible DALY rates for the disease.

**Figure 2:**
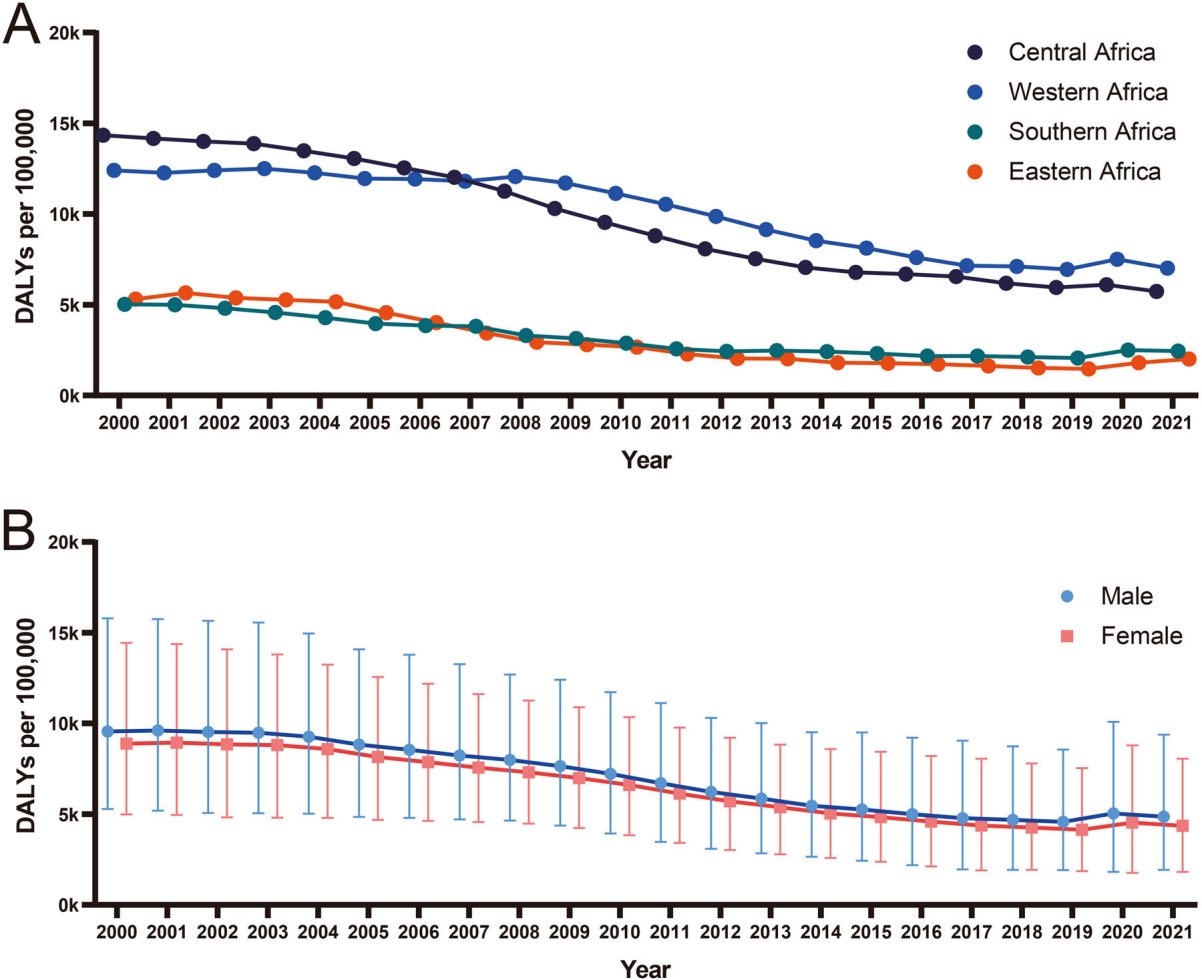
Temporal trends in malaria burden by region and sex in Africa (2000–2021). The line graphs show the annual disability‐adjusted life years (DALYs) per 100,000 for malaria in different African sub‐regions or sexes. (A). DALYs per 100,000 for malaria across all ages and both sexes in different sub‐regions of Africa. (B). DALYs per 100,000 for malaria across all ages in Central Sub‐Saharan Africa (males and females). DALYs data were extracted from the GBD database (https://www.healthdata.org/). Wilcoxon matched‐pairs signed rank test results (Two‐tailed): *p* < 0.0001; Male vs. female across all years (Supporting Information S1: Table 1).

Figure 1 presents a geographical analysis of malaria burden in the year 2021, as indicated by Disability‐adjusted life years (DALYs) per 100,000 population. The data, sourced from the Global Burden of Disease (GBD) database (https://www.healthdata.org/), offers a comprehensive overview of the disease's impact across different regions. A notable observation is the heightened concentration of DALYs in central sub‐Saharan Africa, with countries such as Mali, Niger, Nigeria, the Central African Republic, South Sudan, Chad, Uganda, and the Democratic Republic of Congo recording the highest figures (Figures 1 and 2A). This regional prevalence is indicative of the intense malaria transmission cycles and the associated health challenges in these areas. The stark contrast between central sub‐Saharan Africa and other regions, where the disease burden is comparatively minimal, highlights the uneven nature of malaria's global footprint. In fact, the majority of countries outside of this specific African belt have achieved the elimination of indigenous malaria transmission, with any annual malaria cases primarily consisting of imported infections [61, 62].

There is a clear sex bias in malaria, with a higher prevalence rate in adult males compared to females, as reported by numerous epidemiological studies [63, 64, 65, 66, 67]. We utilized data from the GBD database to analyze the sex differences in malaria burden in Central Sub‐Saharan Africa from the year 2000 to 2021. The results indicated that the malaria mortality burden for females was significantly lower than that for males (Figure 2B).

As early as 1980, the World Health Organization's “Garki Project” (a malaria epidemiological study in West Africa) found that the malaria prevalence rate in males was higher than in females, with lower levels of anti‐malarial parasite antibodies (IHA, IgM) in serum, and this difference was most pronounced in the age groups of 9–18 and 19–28 [66]. Similar conclusions have been drawn from epidemiological surveys in other malaria‐endemic countries or regions. For example, a retrospective analysis of malaria cases in a low‐endemic area in western India (from 2002 to 2007) showed a clear male bias in clinical malaria in Mumbai and Rourkela regions [67]. A nationwide cross‐sectional survey of malaria in Madagascar found that males had a higher prevalence rate in both the adolescent and adult age groups [65]. Another cross‐sectional survey in Brazil also showed a higher prevalence rate in males in almost all age groups (0–10 years, 11–16 years, 16–40 years, and over 40 years) [63]. Epidemiological surveys in Cote d'Ivoire and Ghana also showed a higher overall prevalence rate in males [68, 69]. A retrospective analysis of laboratory‐confirmed malaria cases in China also showed that from 2010 to 2014, the proportion of males (88%) was much greater than that of females [70]. Another population‐based longitudinal surveillance study of malaria in China also showed that the incidence rate in males was much higher than in females (only data with gender information from 2004 to 2013 were included) [64]. However, a recent study in Uganda on the prevalence rate of *Plasmodium falciparum* malaria based on diagnostic confirmation showed that the prevalence rate in females was higher than in males, but the authors believe this may be due to the higher frequency of medical visits by females [71]. Two recent studies from Ghana and Ethiopia also show that males have a higher incidence of malaria than females [72, 73].

Compared to adults, whether there is a sex difference in pediatric malaria is still inconclusive, with varying reports. For example, the aforementioned “Garki Project” results showed no significant gender difference in the prevalence of *P. falciparum* among children under 5 years old. A study in India also showed no difference in the prevalence of malaria between males and females under 10 years old [67]. Another survey in Ghana showed that the prevalence rate in 6–7‐year‐old females was significantly higher than in males [74]. The aforementioned epidemiological survey in Madagascar pointed out that the prevalence rate of malaria was higher in females under 1 year old, while it was higher in males over 2 years old.

The uncertainty of sex differences in pediatric malaria also reflects the role of related hormones in malaria from another perspective, as the differences in hormone levels in the blood of young children are not as pronounced compared to adults.

## 5 Evidence of Estrogen's Impact on Malaria and Its Role in the Disease Process

A multitude of animal experimental results have demonstrated that estrogen can influence malaria: In mouse models of malaria, the administration of exogenous estrogen leads to an increase in the production of IFN‐γ, thereby reducing parasitemia and lowering the incidence rate [75]; however, the opposite effect is observed when estrogen levels are reduced by ovariectomizing the mice [76]. Another study showed that treating female mice or castrated male mice with estrogen reduced their parasitemia and increased the levels of serum TNF‐α [77]. Nevertheless, another study showed that in mice already immune to *Plasmodium chabaudi*, estrogen therapy would not be able to further improve their parasitemia [78]. In a recent study, researchers used the lethal *Plasmodium berghei* ANKA to infect mice, creating an infection model similar to that of human fatal *P. falciparum* malaria. They comprehensively assessed the impact of exogenous estrogen (17β‐estradiol) on malaria through gonadectomy [5]. The results indicated that in intact male mice, 17β‐estradiol treatment significantly increased parasitemia and reduced mouse weight. Concurrently, female mice exhibited higher levels of CD8( + ) T cells and lower levels of NK1.1(+) cells in their spleens compared to male mice. Additionally, gonadectomy increased the levels of IFN‐γ and decreased TNF‐α in the serum of female mice. Notably, infected female and male mice showed significant differences in changes in body temperature and hemoglobin concentration after estrogen treatment. The authors concluded that estrogen triggered a sexually dimorphic pattern in this malaria model in terms of parasitemia, weight changes, body temperature, and hemoglobin concentration. This sexually dimorphic pattern of estrogen during malarial infections may be related to its regulatory effect on oxidative stress. It was found that gonadectomy increased oxidative stress levels in *P. berghei* ANKA‐infected female mice but not in males, suggesting that female sex hormones, particularly estrogens, play a significant role in regulating oxidative stress during malaria infection [79]. Additionally, it has been shown that administration of 17β‐oestradiol differentially modulated oxidative stress depending on sex, infection, and tissue in *P. berghei* ANKA‐infected mice: In the blood, 17β‐oestradiol did not alter superoxide dismutase (SOD) activity but increased catalase (CAT) activity only in intact females, and gonadectomy reduced glutathione peroxidase (GPx) activity in both sexes with 17β‐oestradiol reconstitution only boosting it in females. In the spleen, 17β‐oestradiol increased GPx and CAT activities only in intact males. In the liver, 17β‐oestradiol increased GPx activity only in intact males and decreased CAT activity only in intact males. In the brain, 17β‐oestradiol increased antioxidant enzyme activities only in intact males [80]. These studies suggest that estrogen plays a complex role in the pathophysiology of malaria. The differential effects of estrogen on oxidative stress across different tissues and sexes also highlight the importance of considering hormonal influences in the development of therapeutic strategies for malaria.

Estrogen, via its receptor ERα, has been shown to stimulate the production of type I interferons (IFNs), potentially through the activation of genes involved in innate immune sensing or by directly modulating the expression of type I IFN genes. Type I IFN signaling has been found associated with malaria susceptibility [81, 82]. A series of studies confirm this effect, demonstrating that estrogen enhances the expression of IFN‐responsive genes such as Irf5, Unc93b1, Ifi202 in murine splenocytes [83, 84, 85]. These genes are not only more highly expressed in females under basal conditions but also show increased expression following IFN stimulation [83, 84, 85]. Additionally, estradiol/ERα induces the expression of TRIM‐21 in human monocytes, which in turn increases the stability of IRF3, leading to enhanced production of IFNβ and IL‐23 [86].

Apart from 17β‐estradiol, there have been numerous malaria‐related studies focusing on selective estrogen receptor modulators (SERMs). Tamoxifen, one of the SERMs that is widely used for the treatment of early‐stage breast cancer and to reduce recurrence, has been shown to have a significant inhibitory effect on both in vitro human malaria species *P. falciparum* and in vivo rodent malaria species *P. berghei*, suggesting that long‐term use of this drug may help patients prevent malaria [87]. However, another animal experiment with *P. berghei* ANKA strain infection showed that tamoxifen increased the parasite load in ANKA‐infected mice and exacerbated symptoms by reducing body temperature and weight, while also worsening anemia. Furthermore, tamoxifen significantly increased the splenic index and the percentages of CD4(+) and NK cells and decreased the levels of IL‐2, IL‐6, and IL‐17 in the serum [88]. Therefore, the authors recommend caution when using tamoxifen in breast cancer patients with malaria. There is a difference in the dosage of drugs used in these two animal studies, and sex was not incorporate as a controlled variable within their experimental design. This oversight may have contributed to the seemingly contradictory findings. However, it is important to note that these studies inadvertently provide insight into the potential sexual dimorphism present in malaria. In addition, the antimalarial activities of various SERMs have been specifically evaluated. Among tamoxifen (the first‐generation SERM), raloxifene (the second‐generation SERM), and bazedoxifene (the third‐generation SERM), bazedoxifene exhibited the strongest antimalarial effect and also reduced *P. berghei* infection in female mice, but not in male mice [88]. The IC_50_ concentrations for the inhibition of *P. falciparum* 3D7 by the three were 3.611, 0.761, and 0.160 μM, respectively. Since bazedoxifene is already clinically used and the combination of bazedoxifene‐chloroquine showed a synergistic antiparasitic effect, the authors suggest that bazedoxifene could serve as an effective adjunctive drug to the current antimalarial treatment regimen.

Recent studies have also shed light on the potential of estrogen‐artemisinin hybrids in combating malaria. A review of the role of hormones in malaria infection highlighted the influence on disease severity and therapeutic opportunities, noting the potential of synthetic analogs, receptor agonists, and antagonists of hormones as antimalarial treatments. Specifically, artemisinin‐estrogen hybrids have shown promising in vitro biological activity against *P. falciparum* [89]. Furthermore, the synthesis and evaluation of these hybrids revealed that one particular hybrid was approximately two times more active than artesunate and the standard drug chloroquine, with an EC50 of 3.8 nM, indicating their potential as potent candidates for antimalarial therapy [90]. Additionally, chalcone derivatives on an estradiol framework have been evaluated for their ability to inhibit the growth and development of *P. falciparum*. Several steroidal chalcones with significant antiplasmodial activity were identified, suggesting their potential as antimalarial leads for further optimization and preclinical studies [91]. These studies contribute to the growing body of evidence that estrogen and its derivatives may play a pivotal role in the fight against malaria. The innovative approach of combining estrogen with established antimalarial agents like artemisinin holds promise for developing more effective treatments. As we look ahead, it will be important for future research to explore how these in vitro findings can be translated into clinical applications, which could significantly enhance malaria therapy.

Placental malaria (PM) is a significant complication of pregnancy in malaria‐endemic regions, posing substantial risks to both maternal and fetal health. PM is associated with sequestration of *P. falciparum*‐infected erythrocytes in the placenta, leading to inflammation, oxidative stress, and impairment of placental function [92]. It has been shown that PM significantly impacts levels of sex hormones (including 17β‐estradiol and progesterone) in placental blood, and lower levels of these hormones were associated with increased parasitemia and poor maternal health [93]. During pregnancy, levels of estradiol, estriol, and progesterone increase, which in turn affect transcriptional signaling of inflammatory immune responses at the maternal fetal interface and systemically [94]. Consequently, the reduction in estrogen caused by PM may have profound effects on the maternal immune environment, potentially leading to adverse pregnancy outcomes.

To sum up, in malaria, from the macro‐level surveillance of incidence rates to the micro‐level detection of key molecules in certain signaling pathways, the sexual dimorphism reflected by these changes is closely related to differences in estrogen levels. In fact, women are believed to mount stronger innate and adaptive immune responses, being able to clear infectious diseases faster than men, whether through humoral immunity or cell‐mediated immunity [29]. Compared to men, women exhibit higher activation of immune cells, cytokine production and circulating T lymphocytes [29]. Certainly, in addition to estrogen, other sex hormones including progesterone and testosterone also have direct effects on immune cell function. These hormones are not static; their concentrations fluctuate throughout an individual's life cycle, which can impact parasitic infections such as malaria, as well as bacterial and viral infections. Overall, estrogen can influence the immune system, thereby affecting various stages of malaria.

## 6 Conclusions

**Figure 3:**
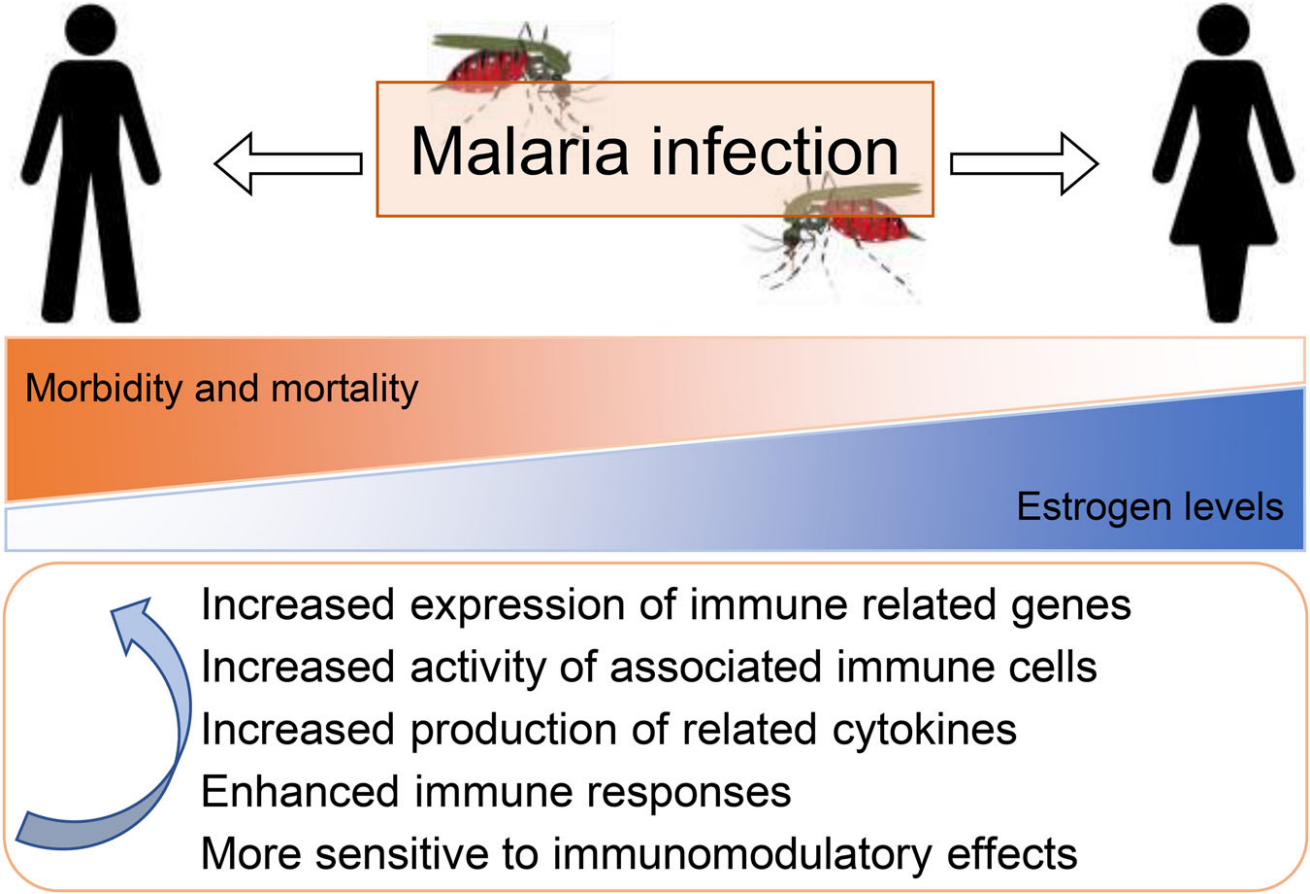
Possible roles of estrogen in sex bias in malaria. Males and females show robust differences in their susceptibility to malaria. Estrogen may affect the human immune response to malaria by enhancing the activation of immune cells, increasing the production of cytokines, and upregulating the expression of immune‐related genes.

The present review provides a focused summary on the role of estrogen in modulating immune responses to malaria (Figure 3), summarizing epidemiological trends and describing relevant animal experimental results, offering a multifaceted view of how estrogen might influence malaria outcomes. The analysis of malaria burden across African regions and sexes over time provides insights into the disease's dynamics and the potential impact of estrogen on these patterns. However, the reliance on existing literature and databases may introduce biases related to data availability and consistency. The cross‐sectional nature of the GBD data limits our ability to infer causal relationships between estrogen levels and malaria outcomes. Additionally, the generalizability of our findings is confined to the regions and demographic groups studied. Variability in methodologies across different studies can affect the comparability of results, and our discussion on the therapeutic implications of SERMs is preliminary due to the absence of specific clinical trials investigating their use in malaria. Further research is needed to address these limitations, including more detailed investigations into the molecular mechanisms of estrogen's action in malaria and clinical trials to assess the efficacy and safety of SERMs as potential therapeutic agents.

Understanding the sex differences in the pathogenesis of malaria is crucial for its prevention, treatment, and vaccine development. Estrogen can modulate the immune responses by regulating the function of various immune cells, thereby affecting malaria, and this action of estrogen may mainly mediated through its receptors ERα and ERβ [8, 13, 24]. Human Immune cells widely express ERα and ERβ, making them target cells for estrogen [17, 18, 19, 20, 21, 22]. Exploring the complexity of estrogen's role in malaria can offer new perspectives for novel therapeutic and vaccination strategies against the disease. Conducting more meticulous and in‐depth investigations in related basic research can contribute to precision medicine or personalized healthcare, which may represent a future direction for research.

## Author Contributions

**Ye Wu:** investigation. **Ying‐Chun Chen:** investigation. **Fang‐Fang Liu:** investigation, writing – original draft preparation, funding acquisition. **Ke Li:** investigation, writing – review and editing, funding acquisition.

## Conflicts of Interest

The authors declare no conflicts of interest.

## Supporting information

Supporting information.

## Data Availability

The authors have nothing to report.

## References

[1] A. A. Lover , J. K. Baird , R. Gosling , and R. N. Price , “Malaria Elimination: Time to Target All Species,” American Journal of Tropical Medicine and Hygiene 99, no. 1 (July 2018): 17–23, 10.4269/ajtmh.17-0869. 29761762 PMC6035869

[2] A. Monroe , N. A. Williams , S. Ogoma , C. Karema , and F. Okumu , “Reflections on the 2021 World Malaria Report and the Future of Malaria Control,” Malaria Journal 21, no. 1 (May 2022): 154, 10.1186/s12936-022-04178-7. 35624483 PMC9137259

[3] H. Bernin and H. Lotter , “Sex Bias in the Outcome of Human Tropical Infectious Diseases: Influence of Steroid Hormones,” supplement, Journal of Infectious Diseases 209, no. S3 (July 2014): S107–S113, 10.1093/infdis/jit610. 24966190

[4] J. Briggs , M. Murray , J. Nideffer , and P. Jagannathan , “Sex‐Linked Differences in Malaria Risk Across the Lifespan,” Current Topics in Microbiology and Immunology 441 (2023): 185–208, 10.1007/978-3-031-35139-6_7. 37695429

[5] L. A. Cervantes‐Candelas , J. Aguilar‐Castro , F. O. Buendía‐González , et al., “17β‐Estradiol Is Involved in the Sexual Dimorphism of the Immune Response to Malaria,” Frontiers in Endocrinology 12 (2021): 643851, 10.3389/fendo.2021.643851. 33841336 PMC8034493

[6] S. M. Liva and R. R. Voskuhl , “Testosterone Acts Directly on CD4+ T Lymphocytes to Increase IL‐10 Production,” Journal of Immunology 167, no. 4 (August 2001): 2060–2067, 10.4049/jimmunol.167.4.2060. 11489988

[7] S. Nilsson , S. Mäkelä , E. Treuter , et al., “Mechanisms of Estrogen Action,” Physiological Reviews 81, no. 4 (October 2001): 1535–1565, 10.1152/physrev.2001.81.4.1535. 11581496

[8] B. Chakraborty , J. Byemerwa , T. Krebs , F. Lim , C. Y. Chang , and D. P. McDonnell , “Estrogen Receptor Signaling in the Immune System,” Endocrine Reviews 44, no. 1 (January 2023): 117–141, 10.1210/endrev/bnac017. 35709009

[9] S. Jaillon , K. Berthenet , and C. Garlanda , “Sexual Dimorphism in Innate Immunity,” Clinical Reviews in Allergy and Immunology 56, no. 3 (June 2019): 308–321, 10.1007/s12016-017-8648-x. 28963611

[10] L. R. Nelson and S. E. Bulun , “Estrogen Production and Action,” supplement, Journal of the American Academy of Dermatology 45, no. S3 (2001): S116–S124, 10.1067/mjd.2001.117432. 11511861

[11] D. W. Brann , Y. Lu , J. Wang , et al., “Brain‐Derived Estrogen and Neural Function,” Neuroscience and Biobehavioral Reviews 132 (January 2022): 793–817, 10.1016/j.neubiorev.2021.11.014. 34823913 PMC8816863

[12] S. Maioli , K. Leander , P. Nilsson , and I. Nalvarte , “Estrogen Receptors and the Aging Brain,” Essays in Biochemistry 65, no. 6 (December 2021): 913–925, 10.1042/EBC20200162. PMC8628183 34623401

[13] N. Fuentes and P. Silveyra , “Estrogen Receptor Signaling Mechanisms,” Advances in Protein Chemistry and Structural Biology 116 (2019): 135–170, 10.1016/bs.apcsb.2019.01.001. 31036290 PMC6533072

[14] Y. S. Xiong , F. F. Liu , D. Liu , et al., “Opposite Effects of Two Estrogen Receptors on Tau Phosphorylation Through Disparate Effects on the miR‐218/PTPA Pathway,” Aging Cell 14, no. 5 (October 2015): 867–877, 10.1111/acel.12366. 26111662 PMC4568974

[15] C. Williams , K. Edvardsson , S. A. Lewandowski , A. Ström , and J. Å. Gustafsson , “A Genome‐Wide Study of the Repressive Effects of Estrogen Receptor Beta on Estrogen Receptor Alpha Signaling in Breast Cancer Cells,” Oncogene 27, no. 7 (February 2008): 1019–1032, 10.1038/sj.onc.1210712. 17700529

[16] D. Song , H. He , R. Indukuri , et al., “ERα and ERβ Homodimers in the Same Cellular Context Regulate Distinct Transcriptomes and Functions,” Frontiers in Endocrinology 13 (2022): 930227, 10.3389/fendo.2022.930227. 35872983 PMC9299245

[17] E. M. Curran , L. J. Berghaus , N. J. Vernetti , A. J. Saporita , D. B. Lubahn , and D. M. Estes , “Natural Killer Cells Express Estrogen Receptor‐α and Estrogen Receptor‐β and Can Respond to Estrogen via a Non‐Estrogen Receptor‐α‐Mediated Pathway,” Cellular Immunology 214, no. 1 (November 2001): 12–20, 10.1006/cimm.2002.1886. 11902825

[18] C. M. Grimaldi , J. Cleary , A. S. Dagtas , D. Moussai , and B. Diamond , “Estrogen Alters Thresholds for B Cell Apoptosis and Activation,” Journal of Clinical Investigation 109, no. 12 (June 2002): 1625–1633, 10.1172/JCI14873. 12070310 PMC151010

[19] S. Laffont , N. Rouquié , P. Azar , et al., “X‐Chromosome Complement and Estrogen Receptor Signaling Independently Contribute to the Enhanced TLR7‐Mediated IFN‐α Production of Plasmacytoid Dendritic Cells From Women,” Journal of Immunology 193, no. 11 (December 2014): 5444–5452, 10.4049/jimmunol.1303400. 25339659

[20] K. Lélu , S. Laffont , L. Delpy , et al., “Estrogen Receptor α Signaling in T Lymphocytes Is Required for Estradiol‐Mediated Inhibition of Th1 and Th17 Cell Differentiation and Protection Against Experimental Autoimmune Encephalomyelitis,” Journal of Immunology 187, no. 5 (September 2011): 2386–2393, 10.4049/jimmunol.1101578. 21810607

[21] K. L. Phiel , R. A. Henderson , S. J. Adelman , and M. M. Elloso , “Differential Estrogen Receptor Gene Expression in Human Peripheral Blood Mononuclear Cell Populations,” Immunology Letters 97, no. 1 (February 2005): 107–113, 10.1016/j.imlet.2004.10.007. 15626482

[22] C. Seillet , S. Laffont , F. Trémollières , et al., “The TLR‐Mediated Response of Plasmacytoid Dendritic Cells Is Positively Regulated by Estradiol In Vivo Through Cell‐Intrinsic Estrogen Receptor α Signaling,” Blood 119, no. 2 (January 2012): 454–464, 10.1182/blood-2011-08-371831. 22096248

[23] D. Kalaitzidis and T. D. Gilmore , “Transcription Factor Cross‐Talk: The Estrogen Receptor and NF‐κB,” Trends in Endocrinology and Metabolism 16, no. 2 (March 2005): 46–52, 10.1016/j.tem.2005.01.004. 15734144

[24] S. E. Dunn , W. A. Perry , and S. L. Klein , “Mechanisms and Consequences of Sex Differences in Immune Responses,” Nature Reviews Nephrology 20, no. 1 (January 2024): 37–55, 10.1038/s41581-023-00787-w. 37993681

[25] N. Kanda , T. Hoashi , and H. Saeki , “The Roles of Sex Hormones in the Course of Atopic Dermatitis,” International Journal of Molecular Sciences 20, no. 19 (September 2019): 4660, 10.3390/ijms20194660. 31547021 PMC6802354

[26] W. Gilmore , L. P. Weiner , and J. Correale , “Effect of Estradiol on Cytokine Secretion by Proteolipid Protein‐Specific T Cell Clones Isolated From Multiple Sclerosis Patients and Normal Control Subjects,” Journal of Immunology 158, no. 1 (January 1997): 446–451. 8977221

[27] S. Kim , S. M. Liva , M. A. Dalal , M. A. Verity , and R. R. Voskuhl , “Estriol Ameliorates Autoimmune Demyelinating Disease: Implications for Multiple Sclerosis,” Neurology 52, no. 6 (April 1999): 1230, 10.1212/wnl.52.6.1230. 10214749

[28] J. G. Zein and S. C. Erzurum , “Asthma Is Different in Women,” Current Allergy and Asthma Reports 15, no. 6 (June 2015): 28, 10.1007/s11882-015-0528-y. 26141573 PMC4572514

[29] S. L. Klein and K. L. Flanagan , “Sex Differences in Immune Responses,” Nature Reviews Immunology 16, no. 10 (October 2016): 626–638, 10.1038/nri.2016.90. 27546235

[30] J. S. Nusbaum , I. Mirza , J. Shum , et al., “Sex Differences in Systemic Lupus Erythematosus,” Mayo Clinic Proceedings 95, no. 2 (February 2020): 384–394, 10.1016/j.mayocp.2019.09.012. 32029091

[31] G. Benedek , J. Zhang , S. Bodhankar , et al., “Estrogen Induces Multiple Regulatory B Cell Subtypes and Promotes M2 Microglia and Neuroprotection During Experimental Autoimmune Encephalomyelitis,” Journal of Neuroimmunology 293 (April 2016): 45–53, 10.1016/j.jneuroim.2016.02.009. 27049561 PMC4824954

[32] K. L. Medina , A. Strasser , and P. W. Kincade , “Estrogen Influences the Differentiation, Proliferation, and Survival of Early B‐Lineage Precursors,” Blood 95, no. 6 (March 2000): 2059–2067. 10706875

[33] S. Subramanian , M. Yates , A. A. Vandenbark , and H. Offner , “Oestrogen‐Mediated Protection of Experimental Autoimmune Encephalomyelitis in the Absence of Foxp3+ Regulatory T Cells Implicates Compensatory Pathways Including Regulatory B Cells: E2 Protection of EAE Without Treg Cells Implicates B Cells,” Immunology 132, no. 3 (March 2011): 340–347, 10.1111/j.1365-2567.2010.03380.x. 21091909 PMC3044900

[34] J. Asaba , M. Bandyopadhyay , M. Kindy , and S. Dasgupta , “Estrogen Receptor Signal in Regulation of B Cell Activation During Diverse Immune Responses,” International Journal of Biochemistry and Cell Biology 68 (November 2015): 42–47, 10.1016/j.biocel.2015.08.012. 26299327

[35] P. L. Härkönen and H. K. Väänänen , “Monocyte‐Macrophage System as a Target for Estrogen and Selective Estrogen Receptor Modulators,” Annals of the New York Academy of Sciences 1089 (November 2006): 218–227, 10.1196/annals.1386.045. 17261769

[36] R. S. Scotland , M. J. Stables , S. Madalli , P. Watson , and D. W. Gilroy , “Sex Differences in Resident Immune Cell Phenotype Underlie More Efficient Acute Inflammatory Responses in Female Mice,” Blood 118, no. 22 (November 2011): 5918–5927, 10.1182/blood-2011-03-340281. 21911834 PMC5363818

[37] S. Kovats , “Estrogen Receptors Regulate an Inflammatory Pathway of Dendritic Cell Differentiation: Mechanisms and Implications for Immunity,” Hormones and Behavior 62, no. 3 (August 2012): 254–262, 10.1016/j.yhbeh.2012.04.011. 22561458 PMC3415586

[38] S. Laffont , C. Seillet , and J. C. Guéry , “Estrogen Receptor‐Dependent Regulation of Dendritic Cell Development and Function,” Frontiers in Immunology 8 (2017): 108, 10.3389/fimmu.2017.00108. 28239379 PMC5300975

[39] E. Carreras , S. Turner , M. B. Frank , et al., “Estrogen Receptor Signaling Promotes Dendritic Cell Differentiation by Increasing Expression of the Transcription Factor IRF4,” Blood 115, no. 2 (January 2010): 238–246, 10.1182/blood-2009-08-236935. 19880499 PMC2808152

[40] F. Xiu , V. C. Anipindi , P. V. Nguyen , et al., “High Physiological Concentrations of Progesterone Reverse Estradiol‐Mediated Changes in Differentiation and Functions of Bone Marrow Derived Dendritic Cells,” PLoS One 11, no. 4 (2016): e0153304, 10.1371/journal.pone.0153304. 27064901 PMC4827838

[41] V. C. Lam and L. L. Lanier , “NK Cells in Host Responses to Viral Infections,” Current Opinion in Immunology 44 (February 2017): 43–51, 10.1016/j.coi.2016.11.003. 27984782 PMC5451301

[42] S. Yang , H. Wang , D. Li , and M. Li , “An Estrogen‐NK Cells Regulatory Axis in Endometriosis, Related Infertility, and Miscarriage,” International Journal of Molecular Sciences 25, no. 6 (March 2024): 3362, 10.3390/ijms25063362. 38542336 PMC10970045

[43] M. Abdullah , P. S. Chai , M. Y. Chong , et al., “Gender Effect on In Vitro Lymphocyte Subset Levels of Healthy Individuals,” Cellular Immunology 272, no. 2 (2012): 214–219, 10.1016/j.cellimm.2011.10.009. 22078320

[44] A. Al‐Attar , S. R. Presnell , C. A. Peterson , D. T. Thomas , and C. T. Lutz , “The Effect of Sex on Immune Cells in Healthy Aging: Elderly Women Have More Robust Natural Killer Lymphocytes Than Do Elderly Men,” Mechanisms of Ageing and Development 156 (June 2016): 25–33, 10.1016/j.mad.2016.04.001. 27059724

[45] S. Chandra , A. K. Tripathi , S. Mishra , M. Amzarul , and A. K. Vaish , “Physiological Changes in Hematological Parameters During Pregnancy,” Indian Journal of Hematology and Blood Transfusion 28, no. 3 (September 2012): 144–146, 10.1007/s12288-012-0175-6. 23997449 PMC3422383

[46] E. J. Molloy , A. J. O'Neill , J. J. Grantham , et al., “Sex‐Specific Alterations in Neutrophil Apoptosis: The Role of Estradiol and Progesterone,” Blood 102, no. 7 (October 2003): 2653–2659, 10.1182/blood-2003-02-0649. 12791649

[47] S. Artham , C. Y. Chang , and D. P. McDonnell , “Eosinophilia in Cancer and Its Regulation by Sex Hormones,” Trends in Endocrinology and Metabolism 34, no. 1 (January 2023): 5–20, 10.1016/j.tem.2022.11.002. 36443206 PMC10122120

[48] M. C. Perez , “Role of Eosinophils in Uterine Responses to Estrogen,” Biology of Reproduction 54, no. 1 (January 1996): 249–254, 10.1095/biolreprod54.1.249. 8838023

[49] Y. Cai , J. Zhou , and D. C. Webb , “Estrogen Stimulates Th2 Cytokine Production and Regulates the Compartmentalisation of Eosinophils During Allergen Challenge in a Mouse Model of Asthma,” International Archives of Allergy and Immunology 158, no. 3 (2012): 252–260, 10.1159/000331437. 22398379

[50] P. J. McKiernan , S. G. J. Smith , A. L. Durham , I. M. Adcock , N. G. McElvaney , and C. M. Greene , “The Estrogen‐Induced miR‐19 Downregulates Secretory Leucoprotease Inhibitor Expression in Monocytes,” Journal of Innate Immunity 12, no. 1 (2020): 90–102, 10.1159/000500419. 31266011 PMC6959121

[51] P. Gourdy , E. A. Bourgeois , A. Levescot , et al., “Estrogen Therapy Delays Autoimmune Diabetes and Promotes the Protective Efficiency of Natural Killer T‐Cell Activation in Female Nonobese Diabetic Mice,” Endocrinology 157, no. 1 (January 2016): 258–267, 10.1210/en.2015-1313. 26485613

[52] A. Stanton , C. Mowbray , M. Lanz , et al., “Topical Estrogen Treatment Augments the Vaginal Response to *Escherichia coli* Flagellin,” Scientific Reports 10, no. 1 (May 2020): 8473, 10.1038/s41598-020-64291-y. 32439855 PMC7242342

[53] J. A. Toonen , A. C. Solga , Y. Ma , and D. H. Gutmann , “Estrogen Activation of Microglia Underlies the Sexually Dimorphic Differences in Nf1 Optic Glioma‐Induced Retinal Pathology,” Journal of Experimental Medicine 214, no. 1 (January 2017): 17–25, 10.1084/jem.20160447. 27923908 PMC5206494

[54] N. Itoh , Y. Itoh , C. E. Meyer , et al., “Estrogen Receptor Beta in Astrocytes Modulates Cognitive Function in Mid‐Age Female Mice,” Nature Communications 14, no. 1 (September 2023): 6044, 10.1038/s41467-023-41723-7. PMC10533869 37758709

[55] A. Hewagama , D. Patel , S. Yarlagadda , F. M. Strickland , and B. C. Richardson , “Stronger Inflammatory/Cytotoxic T‐Cell Response in Women Identified by Microarray Analysis,” Genes and Immunity 10, no. 5 (July 2009): 509–516, 10.1038/gene.2009.12. 19279650 PMC2735332

[56] J. Roved , H. Westerdahl , and D. Hasselquist , “Sex Differences in Immune Responses: Hormonal Effects, Antagonistic Selection, and Evolutionary Consequences,” Hormones and Behavior 88 (February 2017): 95–105, 10.1016/j.yhbeh.2016.11.017. 27956226

[57] R. Shepherd , A. S. Cheung , K. Pang , R. Saffery , and B. Novakovic , “Sexual Dimorphism in Innate Immunity: The Role of Sex Hormones and Epigenetics,” Frontiers in Immunology 11 (2021): 604000, 10.3389/fimmu.2020.604000. 33584674 PMC7873844

[58] S. Kovats , “Estrogen Receptors Regulate Innate Immune Cells and Signaling Pathways,” Cellular Immunology 294, no. 2 (April 2015): 63–69, 10.1016/j.cellimm.2015.01.018. 25682174 PMC4380804

[59] D. Verthelyi and D. M. Klinman , “Sex Hormone Levels Correlate With the Activity of Cytokine‐Secreting Cells In Vivo,” Immunology 100, no. 3 (July 2000): 384–390, 10.1046/j.1365-2567.2000.00047.x. 10929062 PMC2327016

[60] C. McGuinness and K. L. Britt , “Estrogen Receptor Regulation of the Immune Microenvironment in Breast Cancer,” Journal of Steroid Biochemistry and Molecular Biology 240 (June 2024): 106517, 10.1016/j.jsbmb.2024.106517. 38555985

[61] M. Naghavi , K. L. Ong , A. Aali , Collaborators GBDCoD , et al., “Global Burden of 288 Causes of Death and Life Expectancy Decomposition in 204 Countries and Territories and 811 Subnational Locations, 1990‐2021: A Systematic Analysis for the Global Burden of Disease Study 2021,” Lancet 403, no. 10440 (May 2024): 2100–2132, 10.1016/S0140-6736(24)00367-2. 38582094 PMC11126520

[62] J. Mischlinger , C. Rönnberg , M. J. Álvarez‐Martínez , et al., “Imported Malaria in Countries Where Malaria Is Not Endemic: A Comparison of Semi‐Immune and Nonimmune Travelers,” Clinical Microbiology Reviews 33, no. 2 (March 2020), 10.1128/CMR.00104-19. PMC7067581 32161068

[63] L. M. A. Camargo , G. M. D. dal Colletto , M. U. Ferreira , et al., “Hypoendemic Malaria in Rondonia (Brazil, Western Amazon Region): Seasonal Variation and Risk Groups in an Urban Locality,” American Journal of Tropical Medicine and Hygiene 55, no. 1 (July 1996): 32–38, 10.4269/ajtmh.1996.55.32. 8702019

[64] C. Ding , C. Huang , Y. Zhou , et al., “Malaria in China: A Longitudinal Population‐Based Surveillance Study,” Epidemiology and Infection 148 (February 2020): e37, 10.1017/S0950268820000333. 32089144 PMC7058654

[65] T. Kesteman , M. Randrianarivelojosia , C. Mattern , et al., “Nationwide Evaluation of Malaria Infections, Morbidity, Mortality, and Coverage of Malaria Control Interventions in Madagascar,” Malaria Journal 13 (November 2014): 465, 10.1186/1475-2875-13-465. 25431003 PMC4289287

[66] L. Molineaux , J. Storey , J. E. Cohen , and A. Thomas , “A Longitudinal Study of Human Malaria in the West African Savanna in the Absence of Control Measures: Relationships Between Different *Plasmodium* Species, in Particular *P. Falciparum* and *P. Malariae* ,” American Journal of Tropical Medicine and Hygiene 29, no. 5 (September 1980): 725–737, 10.4269/ajtmh.1980.29.725. 6969036

[67] S. Pathak , M. Rege , N. J. Gogtay , et al., “Age‐Dependent Sex Bias in Clinical Malarial Disease in Hypoendemic Regions,” PLoS One 7, no. 4 (2012): e35592, 10.1371/journal.pone.0035592. 22558172 PMC3338423

[68] C. A. Houngbedji , P. B. N'Dri , E. Hürlimann , et al., “Disparities of *Plasmodium falciparum* Infection, Malaria‐Related Morbidity and Access to Malaria Prevention and Treatment Among School‐Aged Children: A National Cross‐Sectional Survey in Côte D'ivoire,” Malaria Journal 14 (January 2015): 7, 10.1186/1475-2875-14-7. 25559587 PMC4326184

[69] K. E. Tiedje , A. R. Oduro , G. Agongo , et al., “Seasonal Variation in the Epidemiology of Asymptomatic *Plasmodium falciparum* Infections Across Two Catchment Areas in Bongo District, Ghana,” American Society of Tropical Medicine and Hygiene 97, no. 1 (July 2017): 199–212, 10.4269/ajtmh.16-0959. PMC5508908 28719306

[70] S. Zhou , Z. Li , C. Cotter , et al., “Trends of Imported Malaria in China 2010–2014: Analysis of Surveillance Data,” Malaria Journal 15 (January 2016): 39, 10.1186/s12936-016-1093-0. 26809828 PMC4727325

[71] J. Okiring , A. Epstein , J. F. Namuganga , et al., “Gender Difference in the Incidence of Malaria Diagnosed at Public Health Facilities in Uganda,” Malaria Journal 21, no. 1 (January 2022): 22, 10.1186/s12936-022-04046-4. 35062952 PMC8778495

[72] D. Hawaria and S. Kibret , “Increased Malaria Incidence Following Irrigation Practices in the Endorheic Rift Valley Basin of Sidama Region, Ethiopia,” PLoS One 18, no. 4 (2023): e0284247, 10.1371/journal.pone.0284247. 37098016 PMC10128979

[73] J. A. Tetteh , P. E. Djissem , and A. K. Manyeh , “Prevalence, Trends and Associated Factors of Malaria in the Shai‐Osudoku District Hospital, Ghana,” Malaria Journal 22, no. 1 (April 2023): 131, 10.1186/s12936-023-04561-y. 37087510 PMC10122813

[74] B. Landgraf , H. Kollaritsch , G. Wiedermann , and W. H. Wernsdorfer , “Parasite Density of *Plasmodium falciparum* Malaria in Ghanaian Schoolchildren: Evidence for Influence of Sex Hormones?,” Transactions of the Royal Society of Tropical Medicine and Hygiene 88, no. 1 (January/February 1994): 73–74, 10.1016/0035-9203(94)90505-3. 8154009

[75] A. Cernetich , L. S. Garver , A. E. Jedlicka , et al., “Involvement of Gonadal Steroids and Gamma Interferon in Sex Differences in Response to Blood‐Stage Malaria Infection,” Infection and Immunity 74, no. 6 (June 2006): 3190–3203, 10.1128/IAI.00008-06. 16714546 PMC1479253

[76] P. W. Klein , J. D. Easterbrook , E. N. Lalime , and S. L. Klein , “Estrogen and Progesterone Affect Responses to Malaria Infection in Female C57BL/6 Mice,” Gender Medicine 5, no. 4 (December 2008): 423–433, 10.1016/j.genm.2008.10.001. 19108815 PMC4155322

[77] R. M. F. Libonati , M. G. Cunha , J. M. Souza , et al., “Estradiol, but Not Dehydroepiandrosterone, Decreases Parasitemia and Increases the Incidence of Cerebral Malaria and the Mortality in *Plasmodium berghei* Anka‐Infected CBA Mice,” Neuroimmunomodulation 13, no. 1 (2006): 28–35, 10.1159/000093271. 16699290

[78] W. P. M. Benten , F. Wunderlich , and H. Mossmann , “ *Plasmodium chabaudi*: Estradiol Suppresses Acquiring, but Not Once‐Acquired Immunity,” Experimental Parasitology 75, no. 2 (September 1992): 240–247, 10.1016/0014-4894(92)90184-c. 1516672

[79] N. A. Mosqueda‐Romo , A. L. Rodríguez‐Morales , F. O. Buendía‐González , M. Aguilar‐Sánchez , J. Morales‐Montor , and M. Legorreta‐Herrera , “Gonadal Steroids Negatively Modulate Oxidative Stress in CBA/Ca Female Mice Infected With *P. berghei* ANKA,” BioMed Research International 2014 (2014): 805495, 10.1155/2014/805495. 25243182 PMC4163401

[80] J. Aguilar‐Castro , L. A. Cervantes‐Candelas , F. O. Buendía‐González , et al., “Dimorphic Effect of 17β‐oestradiol on Pathology and Oxidative Stress in Experimental Malaria,” Immunobiology 225, no. 1 (January 2020): 151873, 10.1016/j.imbio.2019.11.008. 31812344

[81] A. Haque , S. E. Best , M. Montes de Oca , et al., “Type I IFN Signaling in CD8‐ DCs Impairs Th1‐Dependent Malaria Immunity,” Journal of Clinical Investigation 124, no. 6 (June 2014): 2483–2496, 10.1172/JCI70698. 24789914 PMC4038565

[82] G. Samayoa‐Reyes , C. Jackson , S. Ogolla , et al., “IFN‐λ4 Genetic Variants Influence Clinical Malaria Episodes in a Cohort of Kenyan Children,” Malaria Journal 20, no. 1 (April 2021): 196, 10.1186/s12936-021-03689-z. 33882912 PMC8058600

[83] R. Panchanathan , H. Liu , and D. Choubey , “Expression of Murine Unc93b1 Is Up‐Regulated by Interferon and Estrogen Signaling: Implications for Sex Bias in the Development of Autoimmunity,” International Immunology 25, no. 9 (September 2013): 521–529, 10.1093/intimm/dxt015. 23728775 PMC3749904

[84] R. Panchanathan , H. Shen , M. G. Bupp , K. A. Gould , and D. Choubey , “Female and Male Sex Hormones Differentially Regulate Expression of Ifi202, an Interferon‐Inducible Lupus Susceptibility Gene Within the Nba2 Interval,” Journal of Immunology 183, no. 11 (December 2009): 7031–7038, 10.4049/jimmunol.0802665. PMC2783550 19890043

[85] H. Shen , R. Panchanathan , P. Rajavelu , X. Duan , K. A. Gould , and D. Choubey , “Gender‐Dependent Expression of Murine Irf5 Gene: Implications for Sex Bias in Autoimmunity,” Journal of Molecular Cell Biology 2, no. 5 (October 2010): 284–290, 10.1093/jmcb/mjq023. 20802013 PMC2952390

[86] S. Smith , J. Ní Gabhann , E. McCarthy , et al., “Estrogen Receptor α Regulates Tripartite Motif–Containing Protein 21 Expression, Contributing to Dysregulated Cytokine Production in Systemic Lupus Erythematosus,” Arthritis and Rheumatology 66, no. 1 (January 2014): 163–172, 10.1002/art.38187. 24449583

[87] A. Weinstock , J. Gallego‐Delgado , C. Gomes , et al., “Tamoxifen Activity Against *Plasmodium* In Vitro and in Mice,” Malaria Journal 18, no. 1 (November 2019): 378, 10.1186/s12936-019-3012-7. 31775753 PMC6882195

[88] L. Cervantes‐Candelas , J. Aguilar‐Castro , F. Buendía‐González , et al., “Tamoxifen Suppresses the Immune Response to *Plasmodium berghei* ANKA and Exacerbates Symptomatology,” Pathogens 10, no. 6 (June 2021): 743, 10.3390/pathogens10060743. 34204678 PMC8231265

[89] A. Das , M. Suar , and K. S. Reddy , “Hormones in Malaria Infection: Influence on Disease Severity, Host Physiology, and Therapeutic Opportunities,” Bioscience Reports 44, no. 11 (November 2024), 10.1042/BSR20240482. PMC11581842 39492784

[90] T. Fröhlich , A. Kiss , J. Wölfling , et al., “Synthesis of Artemisinin‐Estrogen Hybrids Highly Active Against HCMV, *P. falciparum*, and Cervical and Breast Cancer,” ACS Medicinal Chemistry Letters 9, no. 11 (November 2018): 1128–1133, 10.1021/acsmedchemlett.8b00381. 30429957 PMC6231177

[91] B. S. Sisodia , A. S. Negi , M. P. Darokar , U. N. Dwivedi , and S. P. S. Khanuja , “Antiplasmodial Activity of Steroidal Chalcones: Evaluation of Their Effect on Hemozoin Synthesis and the New Permeation Pathway of *Plasmodium falciparum*‐Infected Erythrocyte Membrane,” Chemical Biology and Drug Design 79, no. 4 (April 2012): 610–615, 10.1111/j.1747-0285.2012.01323.x. 22248242

[92] A. K. Zakama , N. Ozarslan , and S. L. Gaw , “Placental Malaria,” Current Tropical Medicine Reports 7, no. 4 (2020): 162–171, 10.1007/s40475-020-00213-2. 32953387 PMC7493061

[93] R. Megnekou , S. Tenou , J. D. Bigoga , J. C. Djontu , F. M. Medou , and A. Lissom , “Placental Malaria and Modulation of Immune and Hormonal Responses in Cameroonian Women,” Acta Tropica 147 (July 2015): 23–30, 10.1016/j.actatropica.2015.04.001. 25861939

[94] D. P. Robinson and S. L. Klein , “Pregnancy and Pregnancy‐Associated Hormones Alter Immune Responses and Disease Pathogenesis,” Hormones and Behavior 62, no. 3 (August 2012): 263–271, 10.1016/j.yhbeh.2012.02.023. 22406114 PMC3376705

